# Hydrophilic Astragalin Galactoside Induces T Helper Type 1-Mediated Immune Responses via Dendritic Cells

**DOI:** 10.3390/ijms19103120

**Published:** 2018-10-11

**Authors:** Jae Hyoung Jeon, Byung-Cheol Lee, Doman Kim, Daeho Cho, Tae Sung Kim

**Affiliations:** 1 Department of Life Sciences, College of Life Sciences and Biotechnology, Korea University, Seoul 02841, Korea; jjh89_27@naver.com (J.H.J.); microlbc@korea.ac.kr (B.-C.L.); 2 Graduate School of International Agricultural Technology, Seoul National University, Pyeongchang 25354, Korea; kimdm@snu.ac.kr; 3 Institute of Convergence Science, Korea University, Seoul 02841, Korea; cdhkor@korea.ac.kr

**Keywords:** astragalin galactoside, hydrophilic modification, Th1 cell, dendritic cell, adjuvant

## Abstract

A flavonoid Astragalin (kaempferol-3-*O*-β-d-glucopyranoside, Ast) has several biological activities including anti-oxidant, anti-HIV, and anti-allergic effects. Nonetheless, its insolubility in hydrophilic solvents imposes restrictions on its therapeutic applications. In this study, we investigated the effects of water-soluble astragalin-galactoside (kaempferol-3-*O*-β-d-isomaltotrioside, Ast-Gal) on murine bone marrow-derived dendritic cell (DC) maturation and T helper (Th) cell-mediated immune responses. Ast-Gal significantly increased maturation and activation of DCs through the upregulation of surface markers, such as cluster of differentiation (CD)80, CD86, and Major histocompatibility complex (MHC) II in a dose-dependent manner, while Ast had little effects. Additionally, Ast-Gal-treated DCs markedly secreted immune-stimulating cytokines such as interleukin (IL)-1β, IL-6, and IL-12. Importantly, Ast-Gal strongly increased expression of IL-12, a polarizing cytokine of Th1 cells. In a co-culture system of DCs and CD4^+^ T cells, Ast-Gal-treated DCs preferentially differentiates naïve CD4^+^ T cells into Th1 cells. The addition of neutralizing IL-12 monoclonal antibody (mAb) to cultures of Ast-Gal-treated DCs and CD4^+^ T cells significantly decreased interferon (IFN)-γ production, thereby indicating that Ast-Gal-stimulated DCs enhance the Th1 response through IL-12 production by DCs. Injection with Ast-Gal-treated DCs in mice increased IFN-γ-secreting Th1 cell population. Collectively, these findings indicate that hydrophilically modified astragalin can enhance Th1-mediated immune responses via DCs and point to a possible application of water-soluble astragalin-galactoside as an immune adjuvant.

## 1. Introduction

Antigen presenting cells (APCs) are important immune cells that mediate the immune response by presenting foreign antigens. They recognize certain lymphocytes such as T cells complexed with major histocompatibility complexes (MHCs) on their surface. There are several classical APCs, including dendritic cells (DCs), macrophages, Langerhans cells, and B cells, that present foreign antigen to helper T cells interacted with MHC Class II, while others are able to present antigens into the cell to cytotoxic T cells through MHC Class I [1]. Dendritic cells (DCs) are professional antigen-presenting cells (APCs) that play an important role in initiating and controlling immune responses [2]. Tissue-resident immature DCs (iDCs) can capture and process antigens into a small fraction of peptides to be presented on their cell surface with co-stimulatory molecules, but they have a low ability to activate naïve CD4^+^ T cells [3]. When iDCs are activated by relevant stimuli, they become mature DCs and migrate to secondary lymphoid tissues to interact with naïve T cells [4,5]. Activated DCs can present antigens to naïve CD4^+^ T cells by upregulating the MHC II (Signal 1) and co-stimulatory molecules (Signal 2) such as CD80 and CD86. In addition, DCs can be controlled by chemokines and cytokines (Signal 3) [6,7,8,9]. Moreover, the activation of DCs is crucial for the linkage between innate and adaptive immunity.

The activation and differentiation of Th cells, distinguished by the expression of CD4 molecules on their cell surface, are necessary for maintaining and developing immune responses against foreign pathogens [10]. Naïve T cells can be activated by DCs and classified into several subsets depending on their function and cytokines they secrete, including Th1, Th2, Th17, and regulatory T (Treg) cells [10]. Th1 cells usually secrete a large amount of interferon-γ (IFN-γ), which are mainly involved in enhancement of cellular immune responses and for effective elimination of intracellular pathogens such as viruses and bacteria. Th2 cells produce interleukin 4 (IL-4), IL-5, IL-9, IL-10, and IL-13, which are important for pathological implications in asthma and allergic diseases. IL-17-producing Th17 cells are responsible for an effective host defense against extracellular bacteria and fungi [11,12,13]. On the contrary, Treg cells play an essential role in controlling and maintaining immune tolerance via downregulation of effector T cells [14].

Flavonoids are polyphenolic compounds naturally distributed in fruits, vegetables, and plant-based foods and possess potent biological effects including anti-inflammatory, anti-oxidant and anti-carcinogenic activities. Flavonoids exist as either simple or complex glycosides, and humans are estimated to consume approximately 1 g of flavonoids per day. Flavonoids possess a variety of anti-inflammatory properties, some of which are suggested to affect the function of the immune system [15,16,17]. Astragalin (Ast) is a flavonoid extracted from several traditional herbs and medicinal plant components such as green tea seeds, leaves of persimmon, mulberry, and *Rosa agrestis*. Ast has been used to treat many diseases as a traditional Chinese medicine drug for a long time [18]. In the preclinical studies, Ast has shown an inhibitory effect on the production of tumor necrosis factor (TNF)-α, IL-1β, and IL-6 by attenuating activation of the nuclear factor-kappaB cells (NF-κB) signaling pathway [19]. In addition, Ast can reduce acute lung injury in a murine asthma model and enhance survival from lethal endotoxemia [20]. Additionally, Ast has anti-allergic and anti-oxidant activities [21,22]. Nevertheless, the clinical applications of Ast are still limited because it has low water solubility. This drawback results in its slow absorption, inadequate and variable bioavailability, gastrointestinal mucosal toxicity, and delayed development as a prescription drug [23,24]. Recently, astragalin-galactoside (Ast-Gal) became known as an alternative that can overcome this problem [25]. However, the effects of Ast and Ast-Gal on the maturation and activation of DCs are not well studied.

In this study, we demonstrated that hydrophilic Ast-Gal induces phenotypic and functional maturation of DCs, as characterized by the upregulation of the expression of cell surface molecules, and the increased production of immune-stimulating cytokines such as IL-1β, IL-6, and IL-12. Furthermore, Ast-Gal-treated DCs showed increased IFN-γ production and IFN-γ-expressing CD4^+^ T cell population in vitro and in vivo. These data indicate that Ast-Gal induces maturation of DCs to functional APCs, which subsequently differentiate naïve Th cells into IFN-γ-producing Th1 cells.

## 2. Results

### 2.1. Hydrophilic Ast-Gal Induces Expression of Surface Molecules on DCs

**Figure 1:**
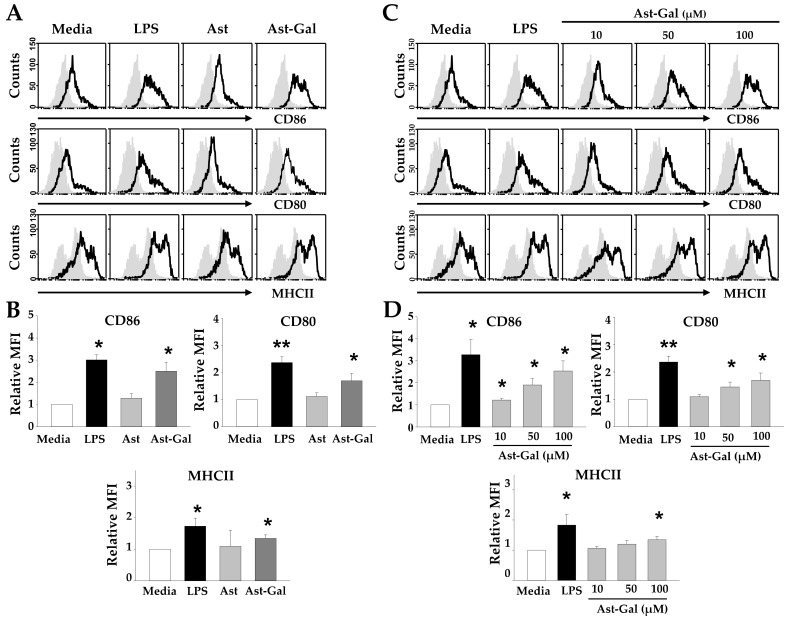
Ast-Gal induces expression of maturation/activation-related surface molecules on DCs. Bone marrow cells were isolated from C57BL/6 mice and differentiated into iDCs, as described in Materials and Methods. (**A**) iDCs (5 × 10^5^ cells/well) were cultured for 18 h in the presence of an indicated concentration of Ast (100 μM), Ast-Gal (100 μM), or lipopolysaccharide (LPS) (100 ng/mL). The expression of CD80, CD86, and MHCII molecules on CD11c^+^ cells was determined by cytofluorometric analysis using PE-conjugated anti-CD80, PE-conjugated anti-CD86, and FITC-conjugated anti-MHC II antibodies, respectively (unshaded areas) or with isotype control antibodies (shaded areas). (**B**) The results from (A) are summarized as mean ± SD (*n* = 3). * *p* < 0.05, ** *p* < 0.01 compared with media-treated DCs. (**C**) iDCs were cultured for 18 h in the presence of various concentrations (10, 50, and 100 µM) of Ast-Gal, or LPS (100 ng/mL). (**D**) The results from (**C**) are summarized as mean ± SD (*n* = 3). The results shown in (**A**) and (**C**) are representative of data from three independent experiments. * *p* < 0.05, ** *p* < 0.01 compared with media-treated DCs.

When immature DCs were activated, they have ability to activate naïve T cell via several signals such as costimulatory molecules, MHC class, and cytokines. Matured DCs have the ability to activate T cell via several signals from costimulatory molecules and MHC class [8]. Nevertheless, iDCs cannot present their antigens to T cell receptor (TCR) because they have insufficient expression of MHC molecules. Although DCs and T cells have a good interaction between antigen-loaded MHC molecules and TCRs, primed T cells hardly progress to effector T cells without costimulatory signal. To test whether Ast-Gal has an effect on the expression of these cell surface molecules, iDCs were incubated for 18 h with Ast or Ast-Gal and the expression of cell surface molecules was determined by cytofluorometric analysis. As shown in Figure 1A,B, Ast had no effect on maturation of DCs. In contrast, Ast-Gal significantly increased the mean fluorescence intensity (MFI) of CD80, CD86, and MHC II on CD11c^+^ DCs. Next, we analyzed whether Ast-Gal dose-dependently increased the MFI of surface markers on DCs. We used different concentrations of Ast-Gal. These results (Figure 1C,D) showed that Ast-Gal upregulated the expression of surface molecules in a dose-dependent manner.

### 2.2. Increased Expression of Immune-Stimulating Cytokines in Ast-Gal-Treated DCs

**Figure 2:**
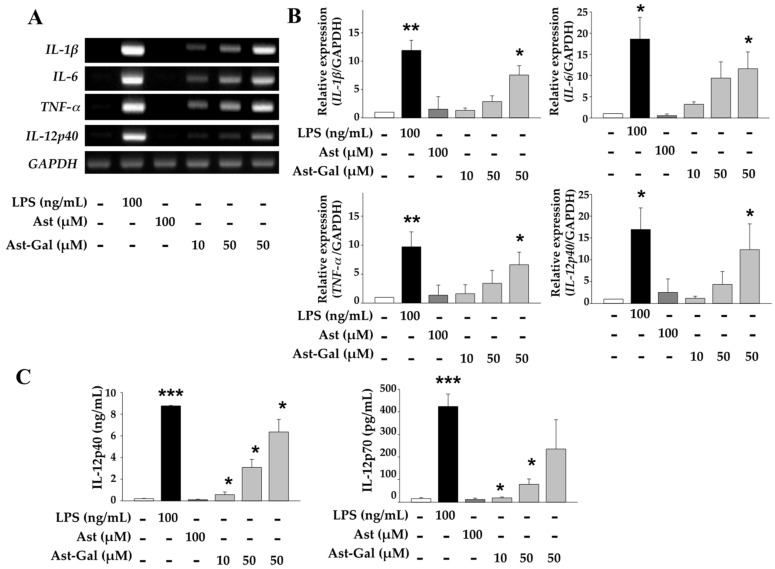
Increased expression of immune-stimulating cytokines in Ast-Gal-treated DCs. (**A**) iDCs (1.5 × 10^6^ cells/well) were cultured for 6 h with various concentrations of Ast-Gal, or Ast (100 uM) or LPS (100 ng/mL) and total RNA was prepared from the dendritic cells. Products of RT-PCR for IL-1β, IL-6, TNF-α, IL-12p40, and GAPDH were analyzed on 1.5% agarose gels. (**B**) The results from (**A**) are summarized as mean ± SD (*n* = 3). * *p* < 0.05, ** *p* < 0.01 compared with media-treated DCs. (**C**) iDCs were treated with various concentrations of Ast-Gal for 24 h, and the levels of IL-12p40 and IL-12p70 in the culture supernatants were determined by a sandwich ELISA. The data are expressed as mean ± SD from three experiments which were conducted in triplicate. * *p* < 0.05, ** *p* < 0.01, *** *p* < 0.001 compared with media-treated DCs.

Maturation and activation of DCs influences the expressional regulation of several genes including cytokine genes. Therefore, we analyzed gene expression of cytokines, especially Th cell-polarizing cytokines such as IL-1β, IL-6, TNF-α, and IL-12 in DCs treated with several concentrations of Ast-Gal, or Ast as a control. As shown in Figure 2A,B, the expression levels of IL-1β, IL-6, TNF-α, and IL-12p40 in Ast-Gal-treated DCs significantly increased as compared to Ast-treated DCs in a dose-dependent manner. To determine whether Ast-Gal can also induce functional maturation of DCs at the protein level, iDCs were treated for 18 h with Ast-Gal or Ast. The levels of IL-12p40 and IL-12p70 proteins in culture supernatants were determined by a sandwich ELISA. Consistent with mRNA levels, Ast-Gal significantly enhanced secretion of IL-12p40 and IL-12p70 in a dose-dependent manner, while Ast did not (Figure 2C). These results clearly indicate the ability of Ast-Gal to mature and activate DCs.

### 2.3. Ast-Gal-Stimulated DCs Enhance IFN-γ Production in CD4^+^ T Cells In Vitro

**Figure 3:**
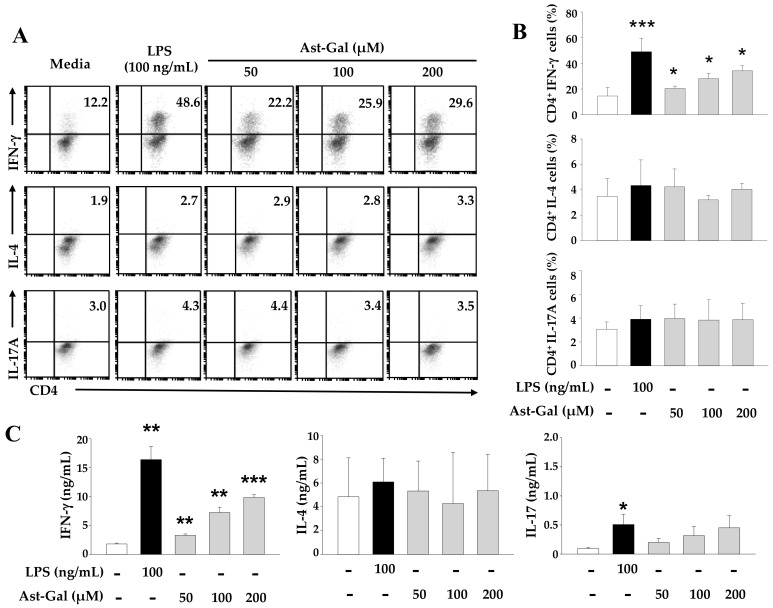
Ast-Gal-stimulated DCs increase IFN-γ production in CD4^+^ T cells in vitro. The iDCs (5 × 10^4^ cells/well) were pulsed with 10 µg/mL of OVA for 2 h, and stimulated for 6 h with various concentrations of Ast-Gal, LPS (100 ng/mL), or media alone (untreated control). Next, untreated and treated DCs were harvested and cocultured with CD4^+^ T cells from OVA-specific OT-II mice at the ratio of 1:10 for 3 days. (**A**) The percentages of IFN-γ-, IL-4-, and IL-17-expressing T cell population were determined by flow cytometric analysis. The results shown are representative of three independent experiments. (**B**) The results from (**A**) are summarized as mean ± SD (*n* = 3). * *p* < 0.05, ** *p* < 0.01, *** *p* < 0.001 compared with media-treated DCs. (**C**) The production levels of IFN-γ, IL-4, and IL-17 were detected by a sandwich ELISA. The data are expressed as mean ± SD from three experiments which were conducted in triplicate. * *p* < 0.05, ** *p* < 0.01, *** *p* < 0.001 compared with media-treated DCs.

Matured DCs are able to induce the polarization of T helper cells toward each subset including Th1, Th2, and Th17. IL-12 is known to have the potential for inducing Th1 cell-mediated responses such as enhancement of IFN-γ production but downregulates Th2 cell- and Th17 cell-mediated responses [9]. Given that Ast-Gal enhanced production of IL-12by DCs, the effect of Ast-Gal-treated DCs on the cytokine profiles of CD4^+^ T cells after co-culture may lead to interesting changes. To investigate whether Ast-Gal-treated DCs can modulate a Th cell-mediated response, ovalbumin (OVA)-pulsed, Ast-Gal-stimulated DCs were co-cultured at a ratio of 1:10 with CD4^+^ T cells. After incubation for 3 days, the cells were collected and then each population subset was confirmed according to the cytokines such as IFN-γ for Th1 cells, IL-4 for Th2 cells, and IL-17A for Th17 cells. As shown in Figure 3A,B, Ast-Gal-treated DCs that were cocultured with OT-II T cells increased IFN-γ production in a dose-dependent manner. In contrast, Ast-Gal did not affect the production of IL-4 and IL-17A in OT-II T cells. Next, we confirmed that the increased percentage of cytokine-producing cells definitely cause greater secretion of Th subset-related cytokines. We analyzed the concentration of each cytokine in supernatants by ELISA. The secretion level of IFN-γ gradually increased with the concentration of Ast-Gal, indicating that Ast-Gal can induce the generation of Th1 cells (Figure 3C). Ast-Gal had statistically negligible effect on Th2 cells and Th17 cells. Furthermore, Ast-Gal did not directly affect the differentiation of CD4^+^ T cells (Figure S1). These results revealed that Ast-Gal enhanced Th1 cell-mediated immune responses via DCs.

### 2.4. IL-12 Secreted from the Ast-Gal-Treated DCs Is Involved in the Increased IFN-γ Production in CD4^+^ T Cells

**Figure 4:**
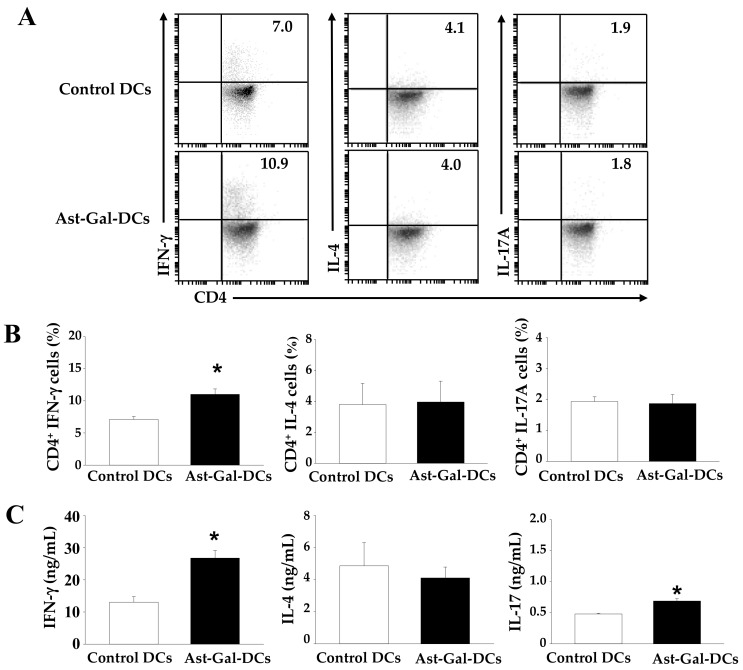
IL-12 secreted by the Ast-Gal-treated DCs is involved in the increased IFN-γ production in CD4^+^ T cells. The iDCs (5 × 10^4^ cells/well) were pulsed with 10 µg/mL OVA for 2 h, and stimulated for 6 h with Ast-Gal (200 μM) or media alone (untreated control). Next, untreated- and treated-DCs were harvested and cocultured with OVA-specific CD4^+^ T cells at the ratio of 1:10 for 3 days in the presence of various concentrations of an anti-IL-12 antibody (0.1, 1, or 10 µg/mL) or isotype control (10 µg/mL). (**A**) The percentages of IFN-γ-expressing T-cell populations were determined by flow cytometric analysis. (**B**) The results from (**A**) are summarized as mean ± SD (*n* = 3). * *p* < 0.05 compared with media-treated DCs. (**C**) The production levels of IFN-γ was detected by a sandwich ELISA. The data are expressed as mean ± SD from three experiments which were carried out in triplicate. * *p* < 0.05, ** *p* < 0.01 compared with media-treated DCs.

Because Ast-Gal-treated DCs exerted the enhanced ability for the induction of IFN-γ production in CD4^+^ T cells in vitro (Figure 3), an anti-IL-12 monoclonal antibody was added to the cell culture to rule out the possibility of the increased IL-12 production by Ast-Gal-treated DCs, which is the main inducer of IFN-γ production. In this study, different concentrations of the anti-IL-12 mAb were added to the culture containing CD4^+^ T cells, and IFN-γ production was analyzed after 3 days of incubation. As shown in Figure 4, the addition of the anti-IL-12 antibody to cultures of Ast-Gal-treated DCs and CD4^+^ T cells significantly increased IFN- γ production. However, the IgG2a isotype control antibody have no effect on the IFN-γ production. These results suggested that IL-12 secretion by Ast-Gal-activated DCs was an essential key for production of IFN-γ by CD4^+^ T cells.

### 2.5. Ast-Gal-Treated DCs Upregulate IFN-γ Production in CD4^+^ T Cells In Vivo

**Figure 5:**
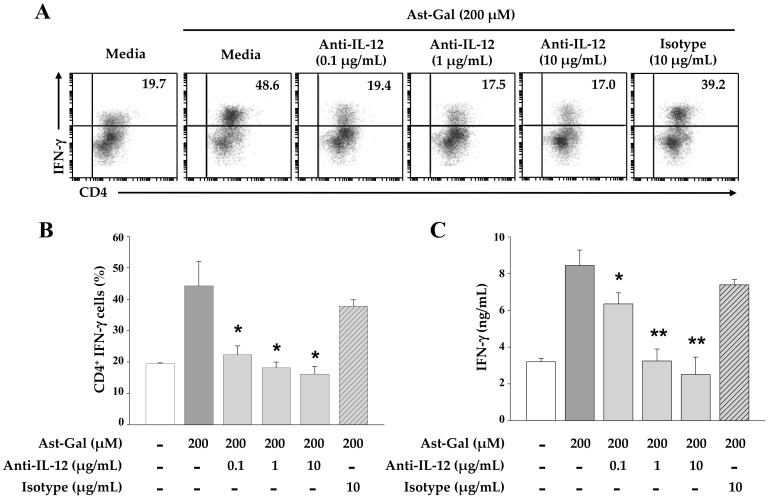
Ast-Gal-treated DCs upregulate IFN-γ production in CD4^+^ T cells in vivo. The iDCs (1 × 10^6^ cells/well) were pulsed with 10 μg/mL of OVA for 2 h. Next, the cells were stimulated for 6 h with Ast-Gal (100 μM) or media alone (control), harvested, and then used to immunize OT-II mice via footpad injection on Day 1 at a total of 1 × 10^6^ DCs per mouse. On Day 7, the lymph node cells were collected and incubated for 3 days in the presence of OVA 100 μg/mL. (**A**) The percentages of IFN-γ-, IL-4-, and IL-17-expressing T cell populations were determined by flow cytometric analysis. (**B**) The results from (**A**) are summarized as mean ± SD (*n* = 3). * *p* < 0.05 compared with media-treated DCs. (**C**) Cell culture supernatants were harvested, and the IFN-γ, IL-4, and IL-17 levels were detected by a sandwich ELISA. The data are expressed as mean ± SD from three independent experiments. * *p* < 0.05 compared with media-treated DCs.

We confirmed that Ast-Gal-treated DCs increased the IFN-γ production by CD4^+^ T cells in vitro. Therefore, we expected that Ast-Gal-treated DCs would also increase IFN-γ production by CD4^+^ T cells in vivo. To investigate whether Ast-Gal-treated DCs induce polarization of T cells in vivo, OT-II mice were immunized by footpad injections on Day 1 with a total of 10^6^ OVA-pulsed DCs that were activated with Ast-Gal for 6 h. Total cells from draining lymph nodes were collected on Day 7 and then incubated for 3 days with OVA. As shown in Figure 5, immunization of mice with Ast-Gal-treated DC significantly induced production of IFN-γ and increased IFN-γ-secreting Th1 cell population. However, production amounts of IL-4 and IL-17, and percentages of IL-4- and IL-17-expressing T-cell populations showed no differences in mice injected with control DCs and Ast-Gal-treated DCs. These results indicated that the stimulatory effect of Ast-Gal-treated DCs significantly enhanced Th1 response in vivo.

## 3. Discussion

Flavonoids are polyphenolic compounds that possess several potent biological functions such as anti-inflammatory, anti-oxidant, and anti-carcinogenic activities [15,16,17]. Astragalin is a flavonoid isolated from several traditional herbs and plant, majorly fond from *R. agrestis* [18]. It has been reported to have various pharmacological effects including anti-oxidant, anti-HIV, anti-allergic, and anti-inflammatory activities [20,21,22]. Although astragalin has one glucosyl unit at the C-3 position of the flavonoid C-ring, it has low water solubility. This shortcoming has restricted clinical applications of astragalin. Lately, astragalin-galactoside was proposed as a solution to the inadequate clinical application [25]. 

This study reveals for the first time that Ast-Gal induces the phenotypic and functional maturation of bone marrow-derived DCs. Ast-Gal significantly increased the expression of several maturation-related surface markers, such as co-stimulatory molecules (CD80 and CD86) and MHC Class II molecule on DCs. At the same time, Ast-Gal increased the expression level of immune-stimulating cytokines, including IL-1 β, IL-6, TNF-α, and IL-12. Furthermore, Ast-Gal-stimulated DCs caused an increase of INF-γ production by the antigen-specific CD4^+^ T cells. Hence, Ast-Gal may induce the differentiation of DCs into the DC1 subset, as indicated by the production of IL-12 and the increase IFN-γ production in CD4^+^ T cells by treatment of Ast-Gal. These results indicate that Ast-Gal may play a role in stimulus for the induction of Th1 cell-mediated immune responses.

Skewed Th1 immune systems are related to effector mechanisms that are important for tumor immunotherapy and some infectious diseases caused by microorganisms. DCs are widely known key mediators of immune responses during inflammation because they perform an important function in adaptive immunity. Furthermore, they control the innate and adaptive immunity by expressing a selective type of Th cell-specific cytokines that determine the balance among Th1, Th2, Th17, or Treg cells [26]. The method for inducing Th1-skewed immune responses by polarized DCs has been developed, and maturated DCs can be used in clinical research on DC-based immunotherapy as an immune adjuvant [27,28]. DCs are usually polarized into DC1 by several activators such as CpGs, LPS, and granulocyte-macrophage colony-stimulating factor (GM-CSF) [29]. It has been reported that DC1 producing large amounts of IL-12 family members are able to rescue patients’ Th1-type anti-melanoma CD4^+^ T-cell responses in vitro [30]. In this study, we propose that Ast-Gal is a differentiation inducer of DC1 cells with Th1-driving ability.

Although Ast-Gal may have effect on cytokine production in CD4^+^ T cells in several ways, IL-12 produced by DCs is an important cytokine for enhancement of IFN-γ secretion by Th1 cells. In an experiment evaluating the blockade of Ast-Gal-stimulated DCs with the neutralizing anti-IL-12 mAb, the production of IFN-γ significantly decreased in CD4^+^ T cells that were coincubated with Ast-Gal-stimulated DCs in the presence of a neutralizing anti-IL-12 mAb. Therefore, IL-12 production by Ast-Gal DCs is a central factor that leads to a Th1 cell-mediated immunity.

In summary, we demonstrated that Ast-Gal may be a crucial controller for inducing the maturation and activation of DCs via upregulation of maturation-associated markers and production of immune-stimulating cytokines, especially IL-12, whereas Ast did not. Moreover, Ast-Gal-treated DCs can differentiate CD4^+^ T cells into IFN-γ-secreting Th1 cells. It is well known that Th1 cells generally control the activation of cytotoxic T lymphocyte. Activated DCs that induce a Th1-meiated immune response can be important for cancer immunotherapy [6]. Hence, Ast-Gal may be able to act as an immune cell vaccine adjuvant in immunotherapy against cancer, for instance, DC-mediated cell therapy.

## 4. Materials and Methods

### 4.1. Experimental Animals

Eight- to 10-week-old C57/BL6 mice (OrientBio, Seongnam, Korea) and ovalbumin (OVA)-specific OT-II TCR Transgenic mice (From Jackson Laboratory, Bar Harbor, ME, USA) were used for this study. The animals were kept in a specific pathogen-free facility at Korea University, Seoul, Korea. All experiments were performed according to the guidelines of Korea University Institutional Animal Care and Use Committee (KUIACUC-2016-174).

### 4.2. Antibodies, Cytokines, and Reagents

Astragalin was modified into astragalin-galactoside (Ast-Gal) using β-galactosidase from *Bacillus circulans*, and purified by the medium pressure chromatography with silica C-18 column followed by Sephadex LH-20 column [25]. Ast-Gal was identified by nuclear magnetic resonance to be kaempferol-3-*O*-β-d-glucopyranosyl-(1->6)-β-d-galactopyranosyl-(1->4)-β-d-galactopyranoside. The water solubility of Ast and Ast-Gal were 28.2 ± 1.2 mg/L and 38,800 ± 2.8 mg/L, respectively. Anti-CD3ε, anti-CD28, and anti-IL-12p70 antibodies were purchased from BD Biosciences (San Diego, CA, USA). Mouse recombinant IFN-γ and IL-4 were purchased from PROSPEC (East Brunswick, NJ, USA). Mouse recombinant IL-6 and TGF-β were purchased from PEPROTECH (Rocky Hill, NJ, USA). FTIC-conjugated anti-CD4, FTIC-conjugated anti-MHC Class II, FTIC-conjugated anti-IgG 2b/k, PE-conjugated anti-IFN-γ, PE-conjugated anti-IL-4, PE-conjugated anti-CD80, PE-conjugated anti-CD86, PerCP-Cy5.5-conjugated anti-CD4, and APC-conjugated anti-IFN-γ antibodies were purchased from BD Biosciences. PE-conjugated anti-Rat IgG2a, PE-conjugated anti-IL-13, APC-conjugated anti-IL-17A, and APC-conjugated anti-CD11c were purchased from eBioscience (San Diego, CA, USA).

### 4.3. Generation of Bone Marrow DCs

The BMDCs were generated via a modified version of the method originally described by Inaba et al. [31]. In brief, the femurs and tibiae of mice were removed and the marrow was flushed with RPMI-1640 with a syringe equipped with 26-gauge needle. Larger cell cluster was dissociated by gentle pipetting and the cell suspension was filtered through a 70 μm nylon cell strainer (BD Falcon, Bedford, MA, USA). Red blood cells were lysed with a lysing solution containing 0.15 M of NH_4_Cl, 1 mM of KHCO_3_, and 0.1 mM of EDTA. The bone marrow cells were then suspended in growth medium. The number of cells in the suspension was then adjusted to 5 × 10^5^ cells/mL, and the cell suspension was added to the culture dishes. The cells were cultured in an RPMI 1640 medium containing heat-inactivated fetal bovine serum, 50 μM 2-ME (Sigma-Aldrich, St Louis, MO, USA), 2 mM glutamine, 1 mM sodium pyruvate, 10 mM HEPES, 100 units/mL penicillin, and 100 μg/mL streptomycin supplemented with 10 ng/mL of granulocyte-macrophage colony-stimulating factor (ProSpec, Rehovot, Israel). The culture medium containing cytokine was replaced on Day 3. At Day 5, the non-adherent cells were removed, and fresh medium containing cytokine was added. At Day 7 of culture, non-adherent cells and loosely adherent DC aggregates were harvested for use in the experiments. 

### 4.4. CD4^+^ T Cell Isolation and T Cell Polarization In Vitro and In Vivo

CD4^+^ T cells were isolated from lymph node and spleen cells using CD4-mircrobeads and MACS system (Miltenyi Biotec, Auburn, CA, USA). CD4^+^ T cells from naïve C57BL/6 mice (1.0 × 10^5^ cells/200 μL) were cultured in antibody (1 μg/mL)-coated 96-well plate with soluble anti-CD28 antibody (1 μg/mL) for (Th1 and Th17 cells) or 3 (Th2 cells) in the presence of Th subset-polarizing cytokines (20 ng/mL mIL-12p70 and 20 ng/mL anti-mIL-4 mAb for Th1; 20 ng/mL mIL-4 and 20 ng/mL anti-IFN-γ mAb for Th2; 20 ng/mL mIL-6, 2 ng/mL hTGF-β1, 20 ng/mL anti-IFN-γ mAb, and 20 ng/mL anti-mIL-4 mAb for Th17).

For the in vitro co-culture assay, CD4^+^ T cells that had been isolated from OT-II mice were cultured at a 10:1 ratio with Ast-Gal-stimulated DCs. Three days later, the cell was thoroughly washed and used for phenotypic and functional characterization, whereas the supernatants were used for cytokine evaluation by ELISA.

For the in vivo assay, OT-II mice were immunized by footpad/haunch injections of 1 × 10^6^ DCs per immunization on Day 1. The cells obtained by draining lymph nodes were collected on Day 7 and cultured for 72 h. The cell was thoroughly washed and used for phenotypic and functional characterization, whereas the supernatants were used for cytokine evaluation by ELISA.

### 4.5. Flow Cytometric Analysis

For cell surface staining, single-cell suspensions were washed and stained in FACS washing buffer (0.5% FBS and 0.05% sodium azide in PBS). For intracellular staining, fixation and intracellular staining were performed in Cytofix/Cytoperm and Perm/Wash solutions (BD Biosciences) according to the manufacturer’s instructions. For the detection of intracellular cytokines, cells were activated by 50 ng/mL poly methyl acrylate and 1 µg/mL ionomycin in the presence of Golgi Plug (BD Biosciences) for 5 h. All of the flow cytometric analyses were performed with gating of live cells using FACSAccuri with BD Accuri C6 software (BD Biosciences).

### 4.6. Reverse Transcriptase-Polymerase Chain Reaction (RT-PCR)

Total RNA that was obtained from the cells was reverse-transcribed into cDNA, and PCR amplification of the cDNA was then performed using a thermal cycler (Bioneer, Daejeon, Korea). The sequences of the PCR primers used in this study were as follows: IL-6 (Fw, 5′ TGA ACA ACG ATG ATG CAC TT 3′; Re, 5′ CGT AGA GAA CAA CAT AAG TC 3′), IL-1β (Fw, 5′ CTA AAG TAT GGG CTG GAC TG 3′; Re, 5′ GGC AGG TCT ACT TTG GAG TC 3′), TNF-α (Fw, 5′ GGC AGG TCT ACT TTG GAG TCA TTG 3′; Re, 5′ ACA TTC GAG GCT CCA GTG AA 3′), IL-12p40 (Fw, 5′ TTA TGC AAA TTG TGA GCT TG 3′; Re, 5′ AGC TTC TTC ATG TCT CCA AA 3′), GAPDH (Fw, 5′ ACA TCA AGA AGG TGG TGA AG 3′; Re, 5′ ATT CAA GAG AGT AGG GAG GG 3′). After amplification, the products were separated on 1.5% (weight/volume) agarose gels and stained with a Staining star (Dyenbio, Kyeongki, Korea).

### 4.7. Enzyme-Linked Immunosorbent Assay (ELISA)

Supernatant was collected from each sample and stored at −80 °C until it was analyzed. The quantities of IL-12p40, IL-12p70, IFN-γ, IL-4, and IL-17A in the culture supernatants were determined via sandwich ELISA using mAbs specific for each cytokine. The mAbs used to coat the plates and the biotinylated second mAb were as follows: for IFN-γ, HB170 and XMG1.2; for IL-12p40, C17.8 and C15.6; for IL-12p70, C18.2 and C17.8; for IL-4, 11B11 and BVD4-1D11; for IL-17A, Mouse IL-17A ELISA kit (eBioscience) according to the manufacturer’s instructions.

### 4.8. Statistical Analysis

All values were expressed as mean ± standard deviation of at least three independent experiments. A paired Student’s *t*-test was used to compare experimental groups with control groups. *p*-values < 0.05 were considered statistically significant.

## Supplementary Materials

The following are available online at http://www.mdpi.com/1422-0067/19/10/3120/s1.

## Abbreviations

Ast	astragalin
Ast-gal	astragalin-galactoside
DC	dendritic cell
Th1	T helper type 1
OVA	ovalbumin
