# Supplementary material for: Hydrophilic Astragalin Galactoside Induces T Helper Type 1-Mediated Immune Responses via Dendritic Cells
Source: Int J Mol Sci. 2018 Oct 11;19(10):3120. doi: 10.3390/ijms19103120 (PMC6213651; doi:10.3390/ijms19103120)
Supplement: Supplementary file 1 [file ijms-19-03120-s001.pdf]

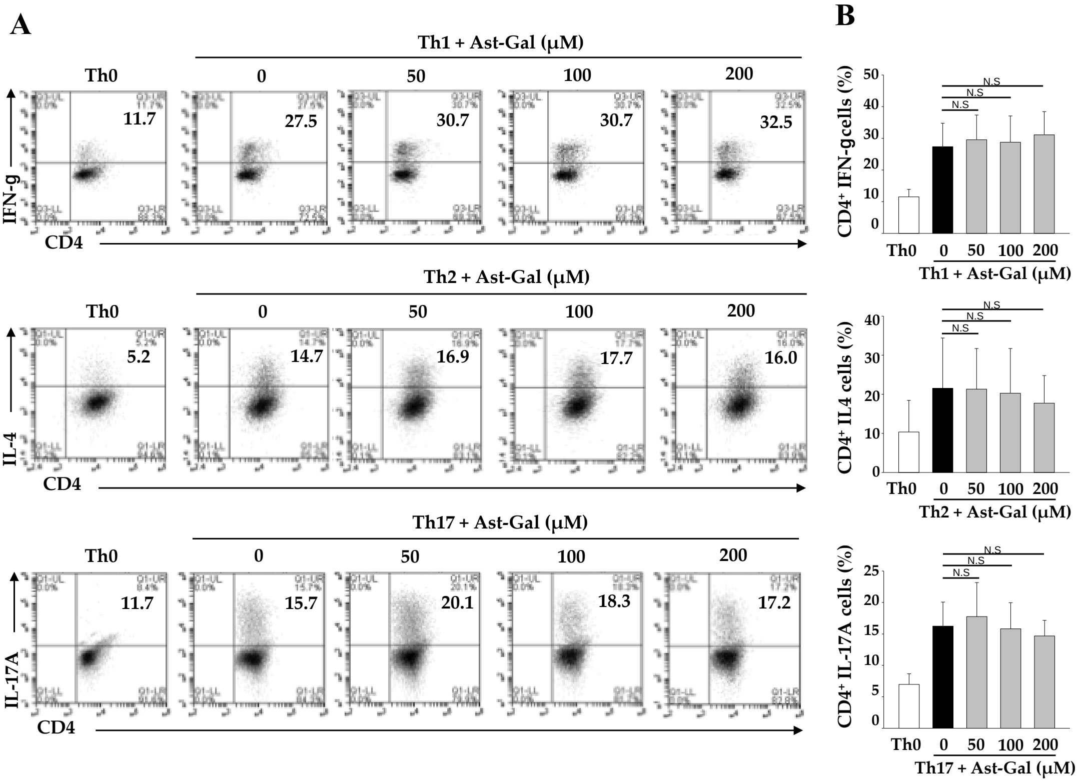


**Figure S1.** Ast-Gal does not directly affect the differentiation of CD4+ T cells. Naïve CD4+ T cells were isolated from C57BL/6 mice and stimulated with anti-CD3 and CD28 mAbs under each Th subset-polarizing condition in the absence or presence of various concentrations of Ast-Gal (0, 50, 100, or 200 μM). (**A**) After 2 or 3 days of incubation, the percentages of cytokine-producing T cell populations was determined by flow cytometric analysis using mAbs for IFN-γ, IL-4, IL-13, and IL-17. (**B**) The results from (A) are summarized as mean ± SD from three independent experiments.
